# Can size distributions of European lake fish communities be predicted by trophic positions of their fish species?

**DOI:** 10.1002/ece3.9087

**Published:** 2022-07-11

**Authors:** Renee M. van Dorst, Christine Argillier, Sandra Brucet, Kerstin Holmgren, Pietro Volta, Ian J. Winfield, Thomas Mehner

**Affiliations:** ^1^ Department of Fish Biology, Fisheries and Aquaculture Leibniz Institute of Freshwater Ecology and Inland Fisheries (IGB) Berlin Germany; ^2^ INRAE, UMR RECOVER Aix‐en‐Provence France; ^3^ Aquatic Ecology Group University of Vic‐Central University of Catalonia Catalonia Spain; ^4^ Catalan Institution for Research and Advanced Studies (ICREA) Barcelona Spain; ^5^ Department of Aquatic Resources, Institute of Freshwater Research Swedish University of Agricultural Sciences Drottningholm Sweden; ^6^ CNR Water Research Institute Verbania Italy; ^7^ Lake Ecosystems Group, UK Centre for Ecology & Hydrology Lancaster Environment Centre Bailrigg UK

**Keywords:** body size, community size spectrum, ecological interactions, fish, predator–prey interactions, trophic level

## Abstract

An organism's body size plays an important role in ecological interactions such as predator–prey relationships. As predators are typically larger than their prey, this often leads to a strong positive relationship between body size and trophic position in aquatic ecosystems. The distribution of body sizes in a community can thus be an indicator of the strengths of predator–prey interactions. The aim of this study was to gain more insight into the relationship between fish body size distribution and trophic position in a wide range of European lakes. We used quantile regression to examine the relationship between fish species' trophic position and their log‐transformed maximum body mass for 48 fish species found in 235 European lakes. Subsequently, we examined whether the slopes of the continuous community size distributions, estimated by maximum likelihood, were predicted by trophic position, predator–prey mass ratio (PPMR), or abundance (number per unit effort) of fish communities in these lakes. We found a positive linear relationship between species' maximum body mass and average trophic position in fishes only for the 75% quantile, contrasting our expectation that species' trophic position systematically increases with maximum body mass for fish species in European lakes. Consequently, the size spectrum slope was not related to the average community trophic position, but there were negative effects of community PPMR and total fish abundance on the size spectrum slope. We conclude that predator–prey interactions likely do not contribute strongly to shaping community size distributions in these lakes.

## Graphical abstract

We aimed to gain more insight in the relationship between fish body size distribution and trophic position in a wide range of European lakes. We had some unexpected results as a strong positive relationship between fish species trophic position and species‐specific maximum body mass is not supported for the fish species inhabiting European lakes. Consequently, the size spectrum slope was not related to the average fish community trophic position in our lakes.

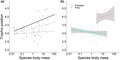

## 1 INTRODUCTION

An organism's body size is one of its most important traits, affecting numerous biological processes such as metabolism, feeding rate, energy use, prey preferences, production, and reproduction (Brown et al., 2004). Consequently, body size plays an important role in ecological interactions such as predation (Peters, 1983). Community size distributions can inform about the state of aquatic ecosystems; for example, they reflect the impacts of water quality (Chu et al., 2016; Cottingham, 1999), climate change (Brucet et al., 2010; Daufresne et al., 2009), fishing (Chu et al., 2016; Graham et al., 2005; Jennings, Greenstreet, et al., 2002), or invasive species (Arranz et al., 2021). Community size distributions can be described as the size spectrum (Kerr & Dickie, 2001; Sheldon et al., 1972), which is a negative linear relationship between the logarithms of body size and the abundance of individuals (power‐law distribution) (White et al., 2007), indicating that abundance decreases with body size, regardless of taxonomy. The slope of the size spectrum indicates the distribution of body sizes in a community; e.g., a shallow slope reflects a community with a relatively high number of large compared to small organisms, while a steeper slope indicates a low abundance of large compared to smaller individuals. Therefore, the slope can be an indicator of the intensity of ecological processes such as predator–prey (Mehner et al., 2016) or competitive interactions (Arranz et al., 2016; Arranz et al., 2019). Furthermore, external influences on aquatic ecosystems such as warming or fishing, which negatively affect in particular large individuals (Daufresne et al., 2009; Law et al., 2016; van Dorst et al., 2019), may also be reflected by a steeper size spectrum slope.

It is commonly assumed for both food web and size spectrum models that there is a strong positive linear correlation between the size of an individual and its trophic position (Brose et al., 2006; Brown et al., 2004; Chang et al., 2014; Kerr & Dickie, 2001). For example, as predators are typically larger than their prey (Cohen et al., 1993; Elton, 1927; Jennings, Pinnegar, et al., 2002; Sheldon et al., 1972), the higher trophic position of predators relative to their prey corresponds to the size difference. The maximum size of prey that can be consumed by a predator increases with the gape size, while simultaneously the metabolic demands increase with predator size (Arim et al., 2007; Mittelbach, 1981; Werner & Gilliam, 1984). Therefore, the trophic position of a species is expected to increase systematically and predictably with body size (Arim et al., 2007; Elton, 1927). Evidence for a positive relationship between species' trophic position and body size comes from aquatic ecosystems (mostly marine fish communities), and a strong positive relationship between trophic position and body size has been found within aquatic communities (Dalponti et al., 2018; France et al., 1998; Gilljam et al., 2011; Reum & Marshall, 2013; Riede et al., 2011), and also for fish species on a global scale (Romanuk et al., 2011). The size structure of (aquatic) food webs is assumed to stem from these strong size‐dependent trophic interactions (e.g., Brose et al., 2006). The decline in abundance of organisms with body size is thus a consequence of energy losses when larger predators feed upon smaller prey (Brown et al., 2004; Kerr & Dickie, 2001). However, some studies have found no evidence at all for a positive relationship between species' body size and trophic position (Jennings et al., 2001; Kopf et al., 2020; Layman et al., 2005), questioning the generality of the concept that larger organisms always occupy higher trophic positions.

If there is indeed a strong relationship between a species' trophic position and its body size, a similarly strong positive relationship between the mean trophic position of communities and their size spectrum slopes can be predicted. For example, as larger fish species are expected to be piscivorous more often and therefore have a higher trophic position (Romanuk et al., 2011), communities characterized by numerous large piscivorous individuals are expected to have a higher mean trophic position than communities dominated by planktivorous or benthivorous individuals. High abundances of piscivores will induce strong predation pressure on small prey fishes. Therefore, these piscivore‐dominated communities would be characterized by shallow size spectrum slopes, which is further conserved because intra‐ and interspecific competition among prey fishes are low and even prey can grow to larger sizes. The mean trophic position and the slope of size distributions should thus be positively correlated among fish communities from several ecosystems.

Another measure of predator–prey interactions that affect the size distribution of a community is the ratio between predator and prey body size (predator–prey mass ratio, PPMR) (Arranz et al., 2019). The realized PPMR (measured from, e.g., gut content of predators) indicates size‐dependent morphological feeding constraints (e.g., gape and handling limitation). For fish, the PPMR is usually around 64 to 125 (Brose et al., 2006), meaning predators are 64 to 125 times heavier (4 to 5 times longer) than their prey. In a community with a high realized PPMR, predators might be able to control the abundance of the prey populations. In turn, in communities with a low PPMR, a substantial proportion of the prey becomes inaccessible to even the largest predators (Mehner et al., 2016). Accordingly, communities with a high PPMR may be characterized by a shallow size spectrum slope, whereas communities with a low realized PPMR should have steeper size distribution slopes due to the weak predation effects from small piscivores (Arranz et al., 2019). However, the realized PPMR is very challenging to measure in studies that attempt to cover such relationships among many ecosystems. Therefore, previous studies often used the community PPMR measured from average body masses of available predators and prey (Arranz et al., 2019; Blanchard et al., 2017; Kerr, 1974). This community PPMR reflects the consequences of size‐specific predator–prey interactions on the distribution of sizes of predator and prey species in the community (Arranz et al., 2019). Overall, we expect a positive relationship between the community PPMR and the size spectrum slope.

Aside from predator–prey interactions, competitive interactions can also affect fish community size spectra. Arranz et al. (2016) showed that an increase in fish abundance (and thereby increased intra‐ and interspecific competition) resulted in a steeper slope of the species‐specific size distribution, for six different fish species common in European lakes. Higher competition for resources likely induces reduced fish growth rates by negative density dependence (e.g., Amundsen et al., 2007; Lorenzen & Enberg, 2002). Decreased growth rates of both prey and predators can lead to a smaller mean body size, a lower size diversity, and an increased number of small compared to large individuals in a community. Low abundances of large predators weaken the control of small prey, maintaining the high abundance and steep size spectrum. These size distributions within a community might thus also reflect the strength of competitive interactions in the community (Arranz et al., 2016), and therefore a negative correlation between the slope of size distributions and the abundance of fishes may be expected.

The aim of this study was to explore the predictors of the slope of fish community size distributions in European lakes. We expanded previous studies by testing whether the slope of size spectra can be predicted by the community trophic position. First, we examined if trophic position systematically increases with maximum body mass for the 48 fish species found in 235 European lakes. Subsequently, we examined if there was a positive relationship between the mean trophic position of the fish communities in these lakes and their size spectrum slopes. In addition, we included PPMR and community abundance as potential predictors of the slope of fish size distributions. Finally, to account for the confounding effects of fish community diversity, lake productivity, temperature, and lake morphometry, which are all known to be predictors of fish size distributions (Emmrich et al., 2011, 2014), we included number of fish species, total phosphorus concentration, maximum annual air temperature, and lake area and maximum depth as covariates in our analyses.

## 2 METHODS

### 2.1 Fish database and sampling

We used the dataset of fish communities in 1943 European lakes and reservoirs accumulated from standardized fishing by multi‐mesh gillnets for the European Water Framework Directive (Brucet et al., 2013; Mehner et al., 2017). Details of background, methods, and basic fish community structure have been summarized earlier (Brucet et al., 2013; Mehner et al., 2017). These lakes were sampled between 1993 and 2012 (from July to early October). Among those lakes, we selected 364 lakes and reservoirs for which complete individual fish size distributions from catches, total number and biomass per species per catch, and several environmental and morphometric variables (maximum temperature, total phosphorus concentration, maximum depth, and lake area) were available. In addition, we used only lakes in which more than 50 fish individuals were caught. The lakes were located in 11 countries (Estonia, France, Germany, Italy, Norway, Slovenia, Spain, Sweden, United Kingdom [England, Scotland, and Wales]), along a latitudinal gradient between 40.8° N and 69.7° N and a longitudinal gradient between 4.6° W and 30.8° E.

A sampling of the fish communities in lakes was done according to the standardized procedure by the European Committee for Standardization (CEN, 2005). Fishes were sampled with benthic multi‐mesh gillnets (length 30 m, height 1.5 m, and 12 mesh‐size panels of 5.5–55 mm) and pelagic multi‐mesh gillnets (length 27.5 m, height 1.5, 3.0, or 6.0 m, and 11 mesh‐size panels between 6.25 and 55 mm), designed to catch the fish representatively with regards to fish abundance, species composition, and size distribution (Appelberg et al., 1995). The European standard was followed for the total numbers of benthic and pelagic nets used per lake, and the distribution of nets across the depth zones. Pelagic nets were only used in lakes with more than 6 m maximum depth. All nets were set in the evening before dusk, left for approximately 12 h, and picked up in the morning after dawn, to include the times when fish are most active. The species of each captured fish was determined and the total length of each fish was measured (rounded to 1 cm). The abundance per species and per 1 cm size class was noted. As not all fishes were weighed individually, we used species‐specific Bayesian length–weight conversion regressions acquired from FishBase (Froese et al., 2014; Froese & Pauly, 2021, extracted in March 2021, see Table S1) to estimate each individual's wet body mass. Only fishes between 8 and 2000 g wet body mass were included in the analyses to control for selectivity of the gill nets, whereby very small‐ and large‐bodied fish are not accurately represented in the catches (similar to Mehner et al. (2016)). The number of species per lake was determined (species richness). Overall, these lakes contained 48 different fish species (see Table S1), but the community composition was dominated (occurrence [number of lakes] and abundance) by the percids perch (*Perca fluviatilis*) and ruffe (*Gymnocephalus cernua*), and the cyprinids roach (*Rutilus rutilus*), common bream (*Abramis brama*), white bream (*Blicca bjoerkna*), and common bleak (*Alburnus alburnus*). Together, these species made up 89.4% of the total number of fishes.

### 2.2 Fish community size spectra

We estimated the individual size distribution of each fish community (lake) using the maximum likelihood estimation (MLE) method (Edwards et al., 2017; White et al., 2008). The MLE method is recommended for calculating continuous size spectra or individual size distributions (Edwards et al., 2017), and describes the shape of the size spectrum independent of the total fish abundance. The individual size distribution can be characterized by a (usually negative) exponent (b) of a probability density function. This b can be estimated from data and is related to the size spectrum slope as obtained from linear regression approaches, where a more negative b corresponds to a steeper slope. Specifically, we used the “MLEbins” method as documented in Edwards et al. (2020), as our fishes were measured in 1 cm size classes. The MLEbins method accounts for the uncertainty in body mass of all the individuals of a species within a length class bin (rather than taking the mean), by assuming that the values within a bin exhibit a power‐law distribution. In addition, this method fully accounts for species‐specific length–weight relationships. The minimum and maximum body mass per bin were estimated using species‐specific length–weight conversion constants extracted from FishBase, and were thus different for each species. Using the abundance in the species‐specific body mass bins, the MLEbins method then finds the value of b that maximizes the likelihood function for each lake and fits a bounded power‐law distribution (also called a truncated Pareto distribution, for more details on the method see Edwards et al., 2020). With our data, we found that the exponent b related well to the slopes between log abundance and log fish size acquired with the traditional least square regression method (*r*[362] = 0.83, *p* < .001), see Figure S1.

**FIGURE 1:**
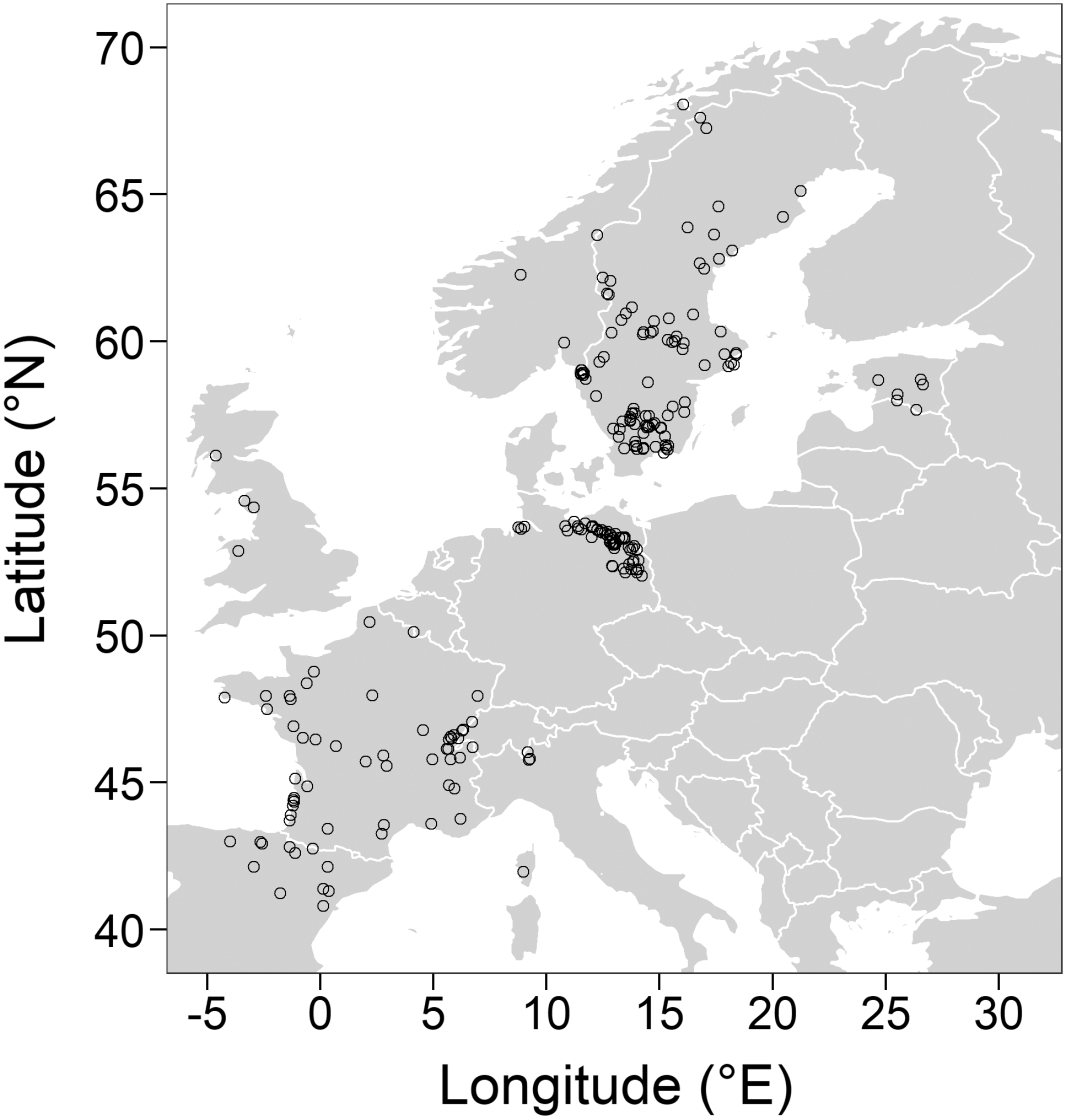
Map showing the distribution of the 235 lakes included in the analyses of our study

There is no statistical approach to evaluate the goodness of fit for the MLE‐based estimation of b. Therefore, we visually assessed the goodness of fit between the empirical and estimated size distributions. We deemed a lake to have an “ill fit” if there was a strong underestimation of small individuals and a strong overestimation of large individuals compared to the empirical data. We performed the analyses only on the lakes that we deemed a good fit (235 of 364 lakes, Figure 1). See Figure S2 for examples of “good” and “ill” fitting MLE's and Figure S3 for a map including all 364 lakes.

### 2.3 Fish community metrics

We extracted fish species‐specific trophic positions from rfishbase (*N* = 48, FoodTroph, R‐package [Boettiger et al., 2012]) and FishBase (Froese & Pauly, 2021). The trophic position in FishBase is estimated from several food items from different studies using a randomized resampling routine. Trophic positions from FishBase are relatively coarse, but previous studies have shown that they correlate well with trophic positions determined from stable isotopes (Carscallen et al., 2012; Mancinelli et al., 2013). Perch, one of the most common species in our database, is known to have strong ontogenetic niche shifts. Therefore, we took a separate trophic position for juvenile (<15 cm) and adult (≥15 cm) perch from individual studies in rfishbase, which correspond to trophic positions found for juvenile and adult perch in other studies (Linzmaier et al., 2018) (for all species‐specific trophic positions, see Table S1). Then, we calculated the arithmetic mean trophic position for each community, weighted by the abundance per species (Table 1).

**TABLE 1:** Overview of the characteristics of the 235 lakes with a “good” MLE fit included in the study. PPMR refers to the predator–prey mass ratio and CPUE refers to the number of catches per unit effort

	Mean	Median	1st quartile	3rd quartile
Trophic position	3.38	3.36	3.17	3.57
PPMR	5.89	4.71	3.29	6.95
CPUE (N net^−1^ night^−1^)	54.75	28	15.48	68.18
Species richness	6.81	7	4	9
Maximum temperature (°C)	16.52	16.70	15.30	17.50
Total phosphorus (μg l^−1^)	36.67	17.8	10.00	41.13
Lake maximum depth (m)	21.64	15	7.95	25.30
Lake area (km^2^)	4.61	1.08	0.51	3.20

The maximum length of each species (*N* = 48) was exacted from rfishbase (Boettiger et al., 2012). The maximum body mass of each species was estimated from species‐specific length–weight conversion constants acquired from FishBase (see Table S1).

We divided all fish into piscivorous predators (from now on “predators”) and prey according to trophic position and body size. All fishes with a trophic position above 3.5 and a body size ≥15 cm were assumed to be predators (15%), and all other fishes were prey (85%) (Table S1). We calculated the predator and prey geometric mean individual body mass of each group per lake. We then calculated the logarithm (log_10_) of the ratio of average predator‐to‐prey body size, and the predator–prey mass ratio (from here PPMR or log_10_(PPMR), Table 1). The PPMR we use is not directly comparable to the realized PPMR from diet data.

Catch per unit effort (CPUE, N net^−1^ night^−1^) per lake was calculated from the total number of fishes caught divided by the number of nets and nights (Table 1). If nets were set in both the benthic and pelagic areas, the mean CPUE over these two habitats was used. To consider the difference in height of pelagic nets, we counted pelagic nets with a height of 1.5 m as equivalent to one standard benthic net (in the net area), while pelagic nets with a height of 3 and 6 m were counted equivalent to two and four standard benthic nets, respectively.

### 2.4 Environmental variables

Environmental predictors included per lake were maximum annual air temperature (°C), lake area (km^2^), lake maximum depth (m), and total phosphorus concentration (total P, μg l^−1^) as obtained from at least four samples across the seasons per year. The lake descriptors were taken from national databases (Table 1).

The maximum air temperature was calculated from the Climatic Research Unit (CRU) model (New et al., 2002), based on temperature records for the years before 2008, thus matching the period when the lakes were sampled. This specific model can obtain a spatial resolution of 10′ latitude and (or) longitude and considers elevational differences between stations (New et al., 2002).

### 2.5 Statistical analyses

To study the effect of species maximum body mass on trophic position, we used quantile regression analyses with trophic position as the response variable and log_10_‐transformed species maximum body mass as the explanatory variable, as quantile regression does not require homoscedasticity of residuals. We were most interested in the median coefficient (50%), and as our data seemed skewed, we also estimated the 25% and 75% quantiles, using the *quantreg* package in R (method = “br”, and se = “boot”). All statistical tests in this study were done in R 4.0.5 (R Core Team, 2017). In addition, we studied the effect of species' maximum body mass on trophic position separately for predators and prey with simple linear regressions.

To study the relationship between the exponent b of the size spectrum and our explanatory variables (trophic position, PPMR, CPUE, species richness, maximum temperature, total phosphorus, maximum depth and lake area) we used a linear mixed effect model. The model did not include random variables, but to account for potential spatial autocorrelation, we added a covariance matrix that depends on the longitude and latitude of the lakes (CorSpatial function (type = exponential) in the *nlme* package (Pinheiro et al., 2021)). To achieve a normal distribution of the residuals, we log_10_ transformed some of the variables. We reviewed variance inflation factor (VIF) values together with a correlation matrix (Table S2) and graphs (Figure S4), and decided all variables can be kept in the model. Finally, we visually inspected the residuals plotted against fitted values to check for normality and homogeneity of variances. In addition, we estimated standardized coefficients to evaluate the effect size of the variables.

## 3 RESULTS

**FIGURE 2:**
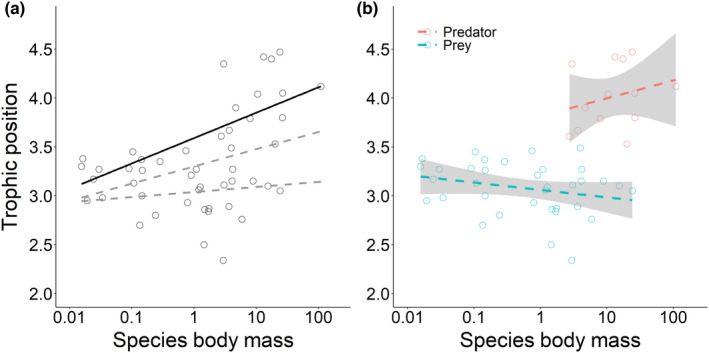
Relationship between trophic position and the maximum body mass (kg) according to FishBase. (a) 25%, 50%, and 75% quantile regression lines. Model output is shown in Table 2. (b) Separate linear regression lines for predators and prey. Solid lines are significant regressions, while dashed lines are not significant

Maximum fish body masses varied between 15.6 g (spined loach, *Cobitis taenia*) and 109.1 kg (Wels catfish, *Siluris glanis*). Trophic position ranged from 2.34 (thinlip mullet, *Chelon ramada*) to 4.47 (asp, *Leuciscus aspius*). Trophic position of fishes increased significantly with a maximum body mass of the 48 species for the 75% quantile, but not for the 25% and 50% quantiles (Table 2, Figure 2a). Maximum body mass of prey varied between 15.6 g (spined loach) and 24.1 kg (common carp, *Cyprinus carpio*), and of predators from 3.0 kg (perch) to 109.1 kg (Wels catfish). For neither prey nor predators was there a significant relationship between species maximum body mass and trophic position (prey: *F*(1,33) = 2.321, *p* = .137, predator: *F*(1,10) = 0.794, *p* = .392, Figure 2b).

**TABLE 2:** Output of the quantile regression analyses for the relationship between species trophic positions and their maximum body mass (*N* = 48) (*p*‐value and a pseudo *R* ^2^)

Quantiles		Value	SE	*t*‐value	*p*‐value	*R* ^2^
25%	(Intercept)	2.884	0.325	8.873		
		0.052	0.102	0.505	.616	0.015
50%	(Intercept)	2.772	0.298	9.288		
		0.176	0.105	1.682	.156	0.046
75%	(Intercept)	2.810	0.190	14.795		
		0.260	0.073	3.583	<.001***	0.243

*Note*: Significance code: *** *p* < .001.

**FIGURE 3:**
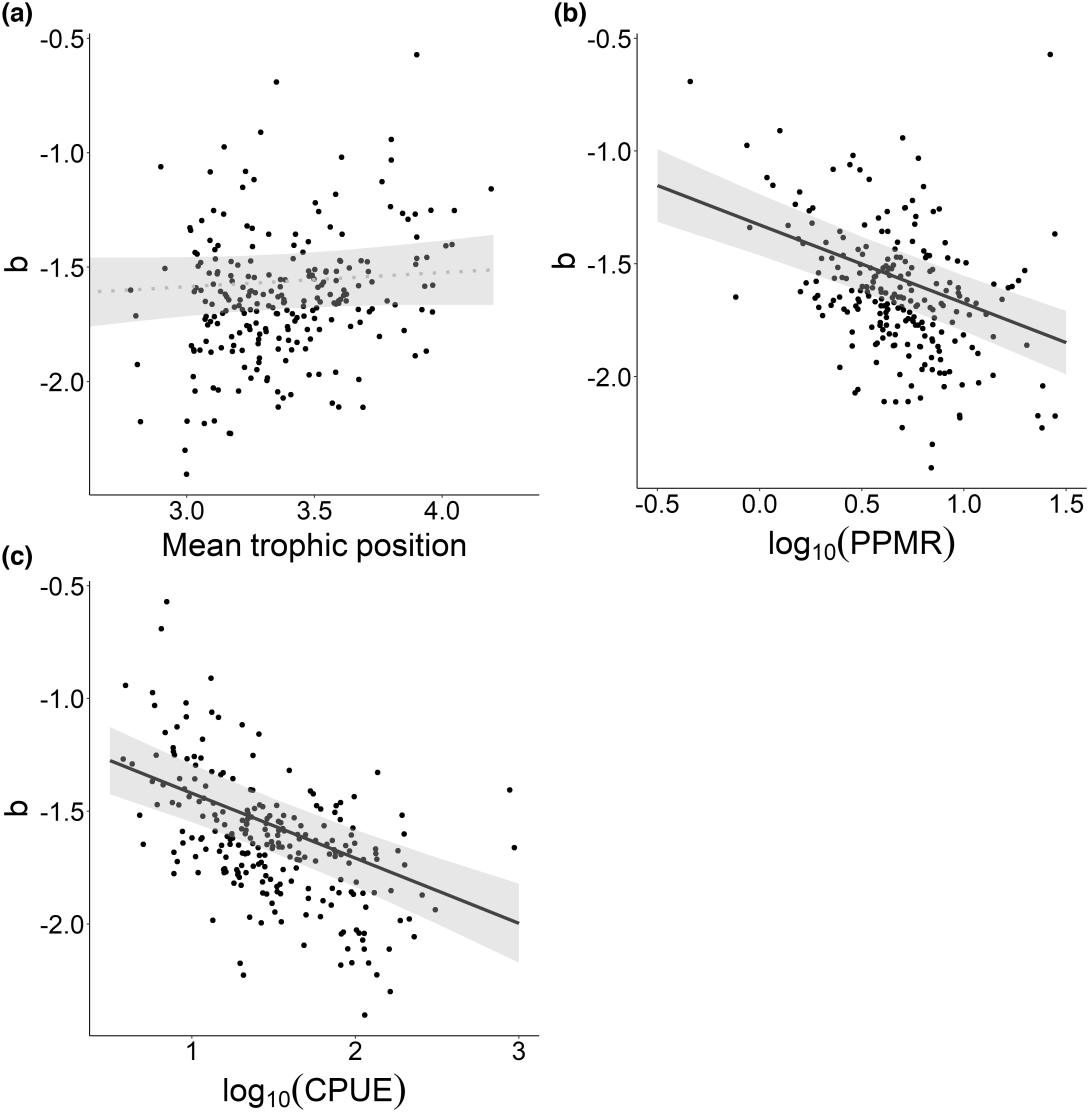
Marginal effect plots from the linear mixed model between the exponent b of the size spectrum and (a) the mean trophic position of a population (averaged over individuals), (b) the ratio of predator‐to‐prey body mass (log_10_(PPMR)), and (c) the catch per unit effort (log_10_(CPUE)). Model outputs are shown in Table 3. Black lines indicate significant relationships with 95% CI intervals, while the gray dotted line represents a non‐significant relationship. *N* = 235 lakes

Variation in the exponent b of the size spectrum among lakes was not significantly affected by the mean trophic position of the fish community (Table 3, Figure 3a). In contrast, we found a negative relationship between log_10_(PPMR) and b (Table 3, Figure 3b), and between log_10_(CPUE) and b (Figure 3c). Changes in CPUE seem to have the largest effect on b (Table 3, standardized coefficients). There was no effect of fish species richness on exponent b (Table 3, Figure S5). Furthermore, none of the environmental variables had a significant effect on b (Table 3, Figure S5).

**TABLE 3:** Output of the model (linear mixed model with a structure to account for potential spatial autocorrelation) relating exponent b of the size spectrum to the mean trophic position of the community, PPMR, CPUE, the species richness, and four environmental covariates

	Value	SE	df	*t*‐value	*p*‐value	Std. value	SE
(Intercept)	−0.734	0.314	226	−2.335	.020*	0.253	0.225
Trophic position	0.061	0.062	226	0.987	.325	0.062	0.062
log_10_(PPMR)	−0.348	0.047	226	−7.447	<.001***	−0.361	0.049
log_10_(CPUE)	−0.288	0.045	226	−6.417	<.001***	−0.457	0.071
Species richness	−0.006	0.007	226	−0.854	.394	−0.064	0.075
Maximum temperature	−0.016	0.010	226	−1.693	.092+	−0.141	0.083
log_10_(Total Phosphorus)	−0.003	0.046	226	−0.065	.949	−0.005	0.077
log_10_(Maximum depth depth)	−0.056	0.050	226	−1.118	.265	−0.080	0.072
log_10_(Area)	0.044	0.027	226	1.645	.101	0.102	0.062

*Note*: In the last two columns, standardized values and errors are noted. *R* ^2^ of the model is 0.40. *N* = 235 lakes. Significance codes: ***p < .001; **p < .01; *p < .05; + *p* < .1.

## 4 DISCUSSION

Our study found a positive linear relationship between maximum body mass and average trophic position on the species level only in the higher quantile (i.e., 75%) for the 48 predominantly freshwater fish species included in this work. This suggests that the common assumption that trophic position increases linearly with body size may not be accurate for fish species in temperate lake systems. Consequently, we also did not find a positive relationship between community trophic position and the exponent b of the size spectrum (slope). However, we unexpectedly found a negative effect of PPMR on exponent b, whereas the strongest predictor of the slope of fish size distributions was CPUE, which had a negative effect on exponent b. This suggests that density‐dependent competitive interactions influence the fish community size distribution more than predator–prey interactions.

As shown by the quantile regression analysis, there was no homogenous positive relationship between species' trophic position and maximum body mass. A significant relationship between trophic position and maximum body size was found only for the 75% quantile among the 48 species. This shows that there is an increase in trophic position with maximum body mass only for fish species with relatively high trophic positions for their body mass. We also see that only species with high maximum body mass (>2.9 kg) achieve high trophic positions qualifying them as piscivorous predators (trophic position>3.5), likely attributable to gape limitation of smaller predators (Kopf et al., 2020). On the other hand, there was no significant change in trophic position with maximum log‐transformed body mass for the 25% and 50% quantiles, indicating that there was no increase in trophic position with maximum body mass for fish species with low and intermediate trophic positions. Thus, we see that fish species with lower trophic levels can have a wide variety in maximum body masses. This is likely because, in addition to small‐sized species, there are also many zooplanktivorous, benthivorous, and omnivorous species with a relatively large maximum body size in European lakes (e.g., *Cyprinus carpio*, *Abramis brama*, and *Coregonus lavaretus*). We thus did not find a strong confirmation for the positive linear relationship between trophic position and log‐transformed maximum body mass as is assumed in size spectrum theory (e.g., Brown et al., 2004; Kerr & Dickie, 2001), and often found empirically (e.g., Dalponti et al., 2018; France et al., 1998; Romanuk et al., 2011). However, other studies have shown similarly that there was no relationship between the trophic position and body size at the species level because fish species with large body sizes often exhibit low trophic positions (Kopf et al., 2020; Layman et al., 2005). Furthermore, we also found no relationship between species trophic position and maximum body mass when assessing predators and prey separately. This finding is in contrast with some studies highlighting a positive relationship between species body size and trophic position when solely carnivorous fish species were included (Keppeler et al., 2020; Riede et al., 2011).

In this study, we assessed the relationship between body size and the average trophic position for 48 frequent species from European lakes as extracted from FishBase. An alternative approach would be to relate the empirically determined maximum size of a species per lake with the empirically determined realized trophic position of this species for each individual lake, based on diet or stable isotope (δ^15^N) data. Such a dataset is difficult to accomplish if a large spatial scale is to be covered because of the enormous amount of data needed both with respect to the within‐lake variability and the variability among lakes. Our results based on the general species‐specific approach contradict the assumption that body sizes of fish species predict their trophic position.

According to the theory that high trophic position coincides with large body mass, a high mean trophic position of the community would be predicted to coincide with a less negative exponent b of the size spectrum, indicating a shallower slope. However, because the trophic position was not correlated systematically with the maximum body mass of fishes in our study, there was also no correlation between the mean trophic position and the size spectrum slope of the fish communities. This is likely partially attributable to the presence of relatively large species (maximum weight >1 kg) with low trophic position (<3.5) in some of our study lakes. On the other hand, one of the most common and abundant predatory species in European lakes is the perch (Thorpe, 1977). Perch has a relatively low average body size in the studied lakes (piscivorous perch [>15 cm]: average 20.1 cm and 138.8 g) but has a relatively high average trophic position (from FishBase = 4.35). In many of the lakes, there were many of these intermediate‐sized perch (± 20 cm) that contributed with their high average trophic position to the mean community trophic position without being a predator capable of strongly feeding upon a larger size range of prey fishes. Accordingly, lakes with a high mean community trophic position (reflecting the high trophic position from piscivorous perch) could nevertheless be characterized by low or intermediate exponents b (reflecting the relatively low size of piscivorous perch). It is therefore not surprising that the relationship between the community trophic position and exponent b of the size spectrum among the lakes was not significant. These results show that, with this dataset, it is difficult to infer the strength of predator–prey interactions from the slope of the size spectrum in European lake fish communities. A more nuanced picture may emerge if the trophic position of species per lake is estimated as average from empirically determined trophic positions, for example, by stable isotope analysis. Empirical estimates would account for potential length‐dependent differences in trophic position within a population (Dalponti et al., 2018; Keppeler et al., 2020). Furthermore, the exact size at which certain species undergo ontogenetic niche shifts may vary between lakes (Hjelm et al., 2000), and hence, for example, the proportion of piscivorous perch may differ between lakes even if the size distributions of perch population would be similar. These differences are not properly covered by using the trophic position attributed to each species by FishBase. However, as outlined above, such a dataset has not been accumulated so far.

Unexpectedly, we found a negative relationship between log_10_(PPMR) and the exponent b of the size spectrum. We had expected that in communities dominated by large predators and small prey (high PPMR), the size distribution would be characterized by a relatively higher proportion of large vs. small fish, reflected by a shallow slope and less negative exponent b. We found the opposite – with increasing PPMR, the exponent b became even more negative. The combination of a shallow size spectrum slope (high b) and a low PPMR is likely caused by a relatively large amount of large prey fish and relatively small predators (e.g., perch of ±20 cm). In these communities, a substantial part of the prey population is in fact larger than the predators, and hence, is unavailable to predators due to gap size limitations. This low fish prey availability may likewise keep the body size of predators small. In turn, communities characterized by high PPMR may contain only few predators (even though they are relatively large). This likely results in weak predation effects and no numerical control of the small prey, with the consequence, that small fishes dominate, and hence the community size spectrum is even steeper than under low PPMR. It is worth mentioning that we did not study the realized PPMR estimated from dietary interactions but estimated PPMR from the numerical presence of predators and prey in the community. Accordingly, the PPMR in the lakes we studied was much lower (mean of 5.8) compared to PPMRs in fish communities accumulated from earlier studies (64–125 (Brose et al., 2006)). The realized PPMRs may be misleading because data from stomach content reflect the size selectivity of the feeding process, not the availability of prey of appropriate size per predator size class. Quantification of mean masses of predators and prey in the total community is a more realistic description of the size differences between predators and prey during encounters. The low PPMR found in many of the lakes suggests that the capabilities of piscivorous fish predators in European lake fish communities to control prey fish communities is extremely limited, as discussed earlier (Mehner, 2010; Mehner et al., 2016).

Due to the sampling method (gill netting) used in this study, fishes with very small (e.g., young‐of‐the year fishes) and very large body sizes (e.g., older adult pike) were not representatively caught (Prchalová et al., 2011). Therefore, we standardized the fish lengths included in the analysis to the range from 8 to 2000 g body mass. To check if our results were affected by the applied size window, we additionally repeated our main analysis by only including fishes with a body mass range between 8 and 1000 g. However, we found only small differences in the exponent b of the size spectrum (Figure S6) and no change in the relationship between b and PPMR (or any of the other variables, Table S3) when using this smaller size window. We, therefore, assume that the underrepresentation of large individuals in gillnet catches has only a weak effect on the estimation of b, and the size selectivity of gillnets is therefore not the reason for the deviations of the results from our previous expectations. Overall, it seems likely that there are no strong predation effects of predatory fishes on their fish prey in European lake communities (Mehner et al., 2016).

Community CPUE had the largest effect size of all explanatory variables and negatively affected the exponent b of the size spectrum. Thus, when CPUE was high, there was a steep size spectrum slope, indicating the dominance of many small and relatively few large individuals. With increasing CPUE, intra‐ and interspecific competition will increase, with lower resource availability per individual fish. Accordingly, stunted growth rates reflecting negative density dependence might become common, leading to a steeper community size spectrum slope. These conclusions are based on findings by Arranz et al. (2016), who demonstrated that the mean size of six species was reduced and the population‐specific size distributions were characterized by a more negative slope with increasing intra‐ and interspecific CPUE of competing individuals. It depends on the PPMR whether the slower‐growing prey individuals at high CPUE become available to predators for longer (within the predation window) or become permanently unavailable when exceeding the maximum gape size of the predators. Here again, it is obvious that the mix of predator–prey and competitive interactions in lake fish communities is both the cause and the consequence of the size distribution of the individuals. From the results accumulated here, it is likely that competition is a stronger structuring force than predation because high abundances of relatively large prey and the dominance of predators with intermediate size like perch often co‐occur in the lakes. However, the expectation that the size distribution and the mean trophic positions of fish communities can inform the relative strengths of these interactions was not supported by the present analysis.

Traditionally, the slope of a community size spectrum is estimated from least‐squares linear regressions between the logarithms of body size and abundance, but lately, the MLE method (based on a power‐law or Pareto distribution) is recommended, as it is more robust (Edwards et al., 2017, 2020). By applying linear regressions to size spectra, strong deviations from linearity have been observed in the size distributions of several fish communities from German lakes (Arranz et al., 2019; Chang et al., 2014). Similar deviations from linearity were identified by using the MLE of continuous size distributions for the fish communities in lakes in our study. About one in three lakes gave a slope that seemed to have an ill fit (visually), and the size distributions from these lakes were therefore excluded from our analyses. The cause of these ill fits seems to be that the proportion of intermediate‐sized fish (around 15 cm) was higher than the proportion of all smaller fish in these lakes. In continuous size distributions (as estimated with the MLE method), each individual gets the same weight for the fitting procedure. The fitted curve is therefore reflecting the most frequent sizes, which sometimes creates strong visual deviations between the empirical and the fitted size distributions. Furthermore, fishes of large sizes are less frequent in all lakes and do not contribute much to the curvature and, hence, to b. These deviations between the individual size distributions and the fitted size spectra are less obvious by applying least‐squares estimation of the slope of a log–log linear regression. It would be interesting for future studies to determine if and how the ill fit of the continuous size distributions is related to both ecological and environmental variables.

Surprisingly, there was no significant correlation between lake environmental variables and exponent b. In several previous analyses focusing on lake fish communities, ecosystem size (depth and area), productivity (total phosphorus and chlorophyll a concentration), geographical location, altitude, and temperature all had a strong‐to‐moderate effect on species mean sizes (Brucet et al., 2013) or the slope of size distributions (Emmrich et al., 2011, 2014). We did not find these effects in the current analysis. In particular, the temperature‐size rule, states that species in warmer lakes have a lower mean population size and a smaller proportion of large individuals, and thus a steeper size spectrum slope (Arranz et al., 2016; Daufresne et al., 2009; van Dorst et al., 2019), could not be demonstrated in the present analysis. These differences in our findings could potentially be due to a difference between exponents b estimated from continuous size distributions and the slope of linear log–log size–abundance regressions. This suggests there might be a conflicting outcome between the rigor to use the most correct statistical approaches to fit size distributions and the ecological implications that are based on more traditional methods of estimating size spectra.

In summary, our results reflect some unexpected findings. First, the assumption that general measures of species' trophic position strongly increase linearly with (log‐transformed) species‐specific maximum body mass was not supported for the fish species inhabiting European lakes. Accordingly, the mean trophic position of fish communities was unrelated to the size distribution of their individuals. In addition, the predator–prey mass ratio was unexpectedly negatively related to the size spectrum slope. Thus, predator–prey interactions likely do not contribute strongly to shaping community size distributions. Finally, we conclude that the method used to fit size distributions needs further scrutinization efforts. The slope fitted with the MLE method showed an ill fit to the data for many of our lakes, raising the question if these deviations between the empirical size distributions and the statistical fit are related to systematic ecological or environmental variabilities between lakes.

## AUTHOR CONTRIBUTIONS

**Renee Van Dorst:** Conceptualization (equal); data curation (equal); formal analysis (lead); methodology (lead); visualization (lead); writing – original draft (lead); writing – review and editing (equal). **Christine Argillier:** Data curation (equal); investigation (equal); resources (equal); writing – review and editing (equal). **Sandra Brucet:** Data curation (equal); investigation (equal); resources (equal); writing – review and editing (equal). **Kerstin Holmgren:** Data curation (equal); investigation (equal); resources (equal); writing – review and editing (equal). **Pietro Volta:** Data curation (equal); investigation (equal); resources (equal); writing – review and editing (equal). **Ian J Winfield:** Data curation (equal); investigation (equal); resources (equal); writing – review and editing (equal). **Thomas Mehner:** Conceptualization (equal); data curation (equal); investigation (equal); methodology (supporting); writing – original draft (supporting); writing – review and editing (equal).

## Supporting information


Appendix S1

## Data Availability

Access to original fishing data is regulated according to Mehner et al. (2017; https://doi.org/10.15504/fmj.2017.23). An overview of the analysed data on lakes used in the manuscript is publicly available at https://doi.org/10.5281/zenodo.6645169.
