# Supplementary material for: Can size distributions of European lake fish communities be predicted by trophic positions of their fish species?
Source: Ecol Evol. 2022 Jul 11;12(7):e9087. doi: 10.1002/ece3.9087 (PMC9272069; doi:10.1002/ece3.9087)
Supplement: Supplementary file 1 — Appendix S1 [file ECE3-12-e9087-s001.pdf]

Supplementary information

Can size spectra of European lake fish communities be predicted by community trophic position?

Renee M. van Dorst[1], Christine Argillier[2], Sandra Brucet[3,4], Kerstin Holmgren[5], Ian J. Winfield[6], Pietro Volta[7], Thomas Mehner[1]

1. Leibniz Institute of Freshwater Ecology and Inland Fisheries (IGB), Department of Fish Biology, Fisheries and Aquaculture, Berlin, Germany
2. INRAE, UMR RECOVER, F-13182, Aix-en-Provence, France
3. University of Vic-Central University of Catalonia, Aquatic Ecology Group, Vic, Spain
4. Catalan Institution for Research and Advanced Studies (ICREA), 08010 Barcelona, Spain
5. Swedish University of Agricultural Sciences, Department of Aquatic Resources, Institute of Freshwater Research, Drottningholm, Sweden
6. Lake Ecosystems Group, UK Centre for Ecology & Hydrology, Lancaster Environment Centre, Bailrigg, Lancaster, United Kingdom
7. CNR Water Research Institute, Verbania, Italy

Table S1 Overview of all fish species found in our lake dataset. For each species we note trophic position (according to FishBase (Froese & Pauly, 2021)), mean length (cm, from our dataset), mean weight (g, calculated from mean length with species-specific conversions), maximum length of species in our dataset (cm, largest individual caught), the maximum weight of the individual with the maximum length (g, calculated from their length with species-specific conversions), the maximum length of the species (cm, according to FishBase), and their maximum weight (g, calculated from their length with species-specific conversions), the number of lakes a species was caught in (N lakes) and the total number of individuals of this species in our dataset (N ind.), and the species-specific length-weight conversion coefficients (from FishBase (Froese & Pauly, 2021)).

Species	Common name	Trophic position	Predator/ prey	Mean length	Mean weight	Max. length caught	Max. weight caught	Max. length FishBase	Max. weight FishBase	N lakes	N ind.	a	b
Abramis brama	Common bream	3.15	Prey	18.6	164	50	1942	82	8900	129	8228	0.00871	3.14
Alburnus alburnus	Common bleak	2.70	Prey	12.0	17	29	225	25	135	108	8108	0.00646	3.09
Ameiurus melas	Black bullhead	3.49	Prey	13.3	39	28	291	66	3970	15	3886	0.00871	3.11
Anguilla anguilla	European eel	3.67	Predator	52.5	365	93	1605	121.5	3673	10	11	0.00095	3.16
Ballerus ballerus	Zope/blue bream	3.21	Prey	27.6	340	40	936	40	900	1	28	0.00871	3.13
Barbatula barbatula	Stone loach	3.45	Prey	9.1	10	10	13	21	105	3	8	0.01	3.04
Barbus barbus	Common barbel	3.10	Prey	46.7	1002	59	1877	120	15396	2	7	0.00891	3
Barbus haasi	Catalonian barbel	3.35	Prey	21.0	104	21	104	30	286	1	1	0.00955	3.03
Blicca bjoerkna	Silver bream	3.09	Prey	14.4	57	50	1743	45.5	1258	89	12181	0.00813	3.13
Carassius auratus	Goldfish	2.86	Prey	23.2	391	42	1036	48	1492	2	9	0.01349	3
Carassius carassius	Crucian carp	3.11	Prey	28.8	364	44	1028	64	3037	10	44	0.01259	2.98
Carassius gibelio	Prussian carp	2.50	Prey	37.0	755	37	755	46.6	1451	1	1	0.0138	3.01
Chelon auratus	Golden grey mullet	2.84	Prey	49.0	1014	49	1014	59	1705	1	1	0.00977	2.96
Chelon ramada	Thinlip mullet	2.34	Prey	47.9	1010	58	1731	70	2950	2	67	0.00977	2.97
Cobitis taenia	Spined loach	3.30	Prey	11.0	10	11	10	13.5	16	1	1	0.0049	3.1
Coregonus albula	Vendace	3.06	Prey	15.3	40	29	250	48	1194	33	1377	0.00479	3.21
Coregonus lavaretus	Whitefish	3.15	Prey	23.3	184	55	1706	73	4101	32	727	0.00447	3.2
Cottus gobio	European bullhead	3.28	Prey	8.0	8	8	8	18	90	1	1	0.00977	3.16
Cottus poecilopus	Alpine bullhead	2.98	Prey	10.5	13	12	19	15	34	1	24	0.00708	3.13
Cyprinid hybrid		3.00	Prey	15.2	83	40	991	NA	NA	24	119	0.01862	2.94
Cyprinus carpio	Common carp	3.05	Prey	29.5	693	50	1895	120	24142	17	266	0.01862	2.94
Esox lucius	European pike	4.40	Predator	40.3	540	66	1875	137	17496	150	440	0.00437	3.09
Gasterosteus aculeatus	Three-spined stickleback	3.38	Prey	9.0	11	9	11	11	16	1	1	0.01047	3.07
Gobio gobio	Gudgeon	3.13	Prey	11.8	22	26	223	21	108	19	766	0.00759	3.14
Gobio lozanoi	Iberian gudgeon	3.17	Prey	9.6	10	12	19	13.5	24	3	184	0.00741	3.11
Gymnocephalus cernua	Eurasian ruffe	3.26	Prey	10.2	12	21	94	25	148	126	7920	0.00977	2.99
Lepomis gibbosus	Pumpkinseed	3.27	Prey	10.2	20	19	117	40	1087	23	401	0.01175	3.1

Leuciscus aspius	Asp	4.47	Predator	32.8	591	47	1489	120	24002	4	5	0.01389	3
Leuciscus idus	Ide	3.79	Predator	20.0	210	44	1055	85	7944	3	7	0.00759	3.12
Leuciscus leuciscus	Common dace	2.93	Prey	21.4	156	31	375	40	791	4	21	0.00794	3.12
Leucos aula		2.80	Prey	10.2	16	16	57	26	242	3	411	0.0081	3.16
Lota lota	Burbot	4.05	Predator	30.0	270	65	1961	152	26438	23	123	0.00479	3.09
Luciobarbus graellsii		2.76	Prey	27.3	310	55	1917	80	5910	7	283	0.00813	3.08
Micropterus salmoides	Largemouth bass	4.42	Predator	18.5	110	32	459	97	13162	7	63	0.01047	3.07
Oncorhynchus mykiss	Rainbow trout	3.53	Predator	31.2	372	47	1149	122	20030	6	24	0.00955	3.03
Osmerus eperlanus	European smelt	3.46	Prey	11.9	13	21	71	45	743	16	477	0.00427	3.17
Parachondrostoma miegii		3.00	Prey	14.0	32	30	270	25	146	9	2213	0.00724	3.08
Perca fluviatilis	European perch	3.78/4.35	Predator	10.7/20.1	18/139	50	1763	60	2997	213	68834	0.01	3.08
Phoxinus phoxinus	Eurasian minnow	3.27	Prey	9.5	11	11	16	14	30	5	16	0.00794	3.12
Rhodeus amarus	European bitterling	2.95	Prey	8.0	8	8	8	11.2	19	1	1	0.01023	3.12
Rutilus rutilus	Common roach	2.87	Prey	13.7	46	43	1108	50.2	1738	206	65768	0.00794	3.14
Salmo trutta	Brown trout	3.80	Predator	26.0	263	59	1991	140	26383	24	587	0.00871	3.02
Salvelinus umbla	Lake char	3.90	Predator	18.0	158	56	1989	75	4692	10	1601	0.00977	3.03
Sander lucioperca	Zander/pikeperch	4.04	Predator	23.5	271	58	1988	100	10476	74	2227	0.00661	3.1
Scardinius erythrophthalmus	Common rudd	2.89	Prey	17.1	113	43	1206	61.7	3627	102	3222	0.00832	3.15
Silurus glanis	Wels catfish	4.12	Predator	39.4	441	55	1025	273	109088	8	19	0.00794	2.93
Squalius cephalus	Common chub	3.61	Predator	26.2	421	53	1916	NA	NA	15	273	0.00776	3.12
Telestes souffia	Souffia	3.37	Prey	13.9	29	20	78	25	144	2	105	0.00692	3.09
Tinca tinca	Tench	3.27	Prey	32.6	697	54	1967	70	4190	57	169	0.01122	3.02

Table S2 Pearson correlations between all variables in the original model. All values were below 0.6, suggesting no strong correlations between explanatory variables. N = 235.

	log_{10} (PPMR)	log_{10} (CPUE)	Species richness	Max T	log_{10} (Total P)	log_{10} (Max Depth)	log_{10} (Area)
**Trophic position**	-0.12	-0.38	-0.45	-0.53	-0.48	0.10	-0.16
**log_{10}(PPMR)**		0.12	-0.09	-0.13	-0.07	-0.08	0.01
**log_{10}(CPUE)**			0.31	0.39	0.53	-0.49	0.12
**Species richness**				0.50	0.58	0.15	0.45
**Max T**					0.52	0.02	0.29
**log_{10}(Total P)**						-0.27	0.17
**log_{10}(Max Depth)**							0.41

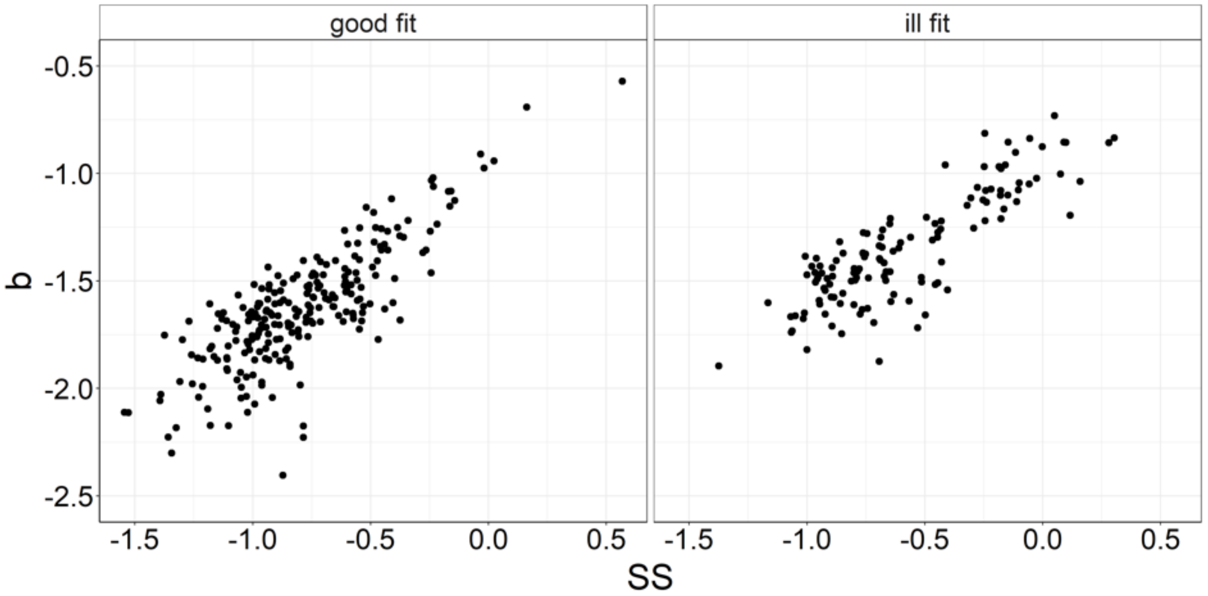

Figure S1 Comparison between the slopes based on maximum likelihood (MLE, as used in the study) and the ordinary least squares (OLS) approach. OLS slopes were based on binning of size classes and calculated with linear regressions. Both for good fit lakes (r(233) = 0.81, p < 0.001) and ill fit lakes (r(127) = 0.84, p < 0.001) there was a positive correlation between the slopes based on the two different methods.

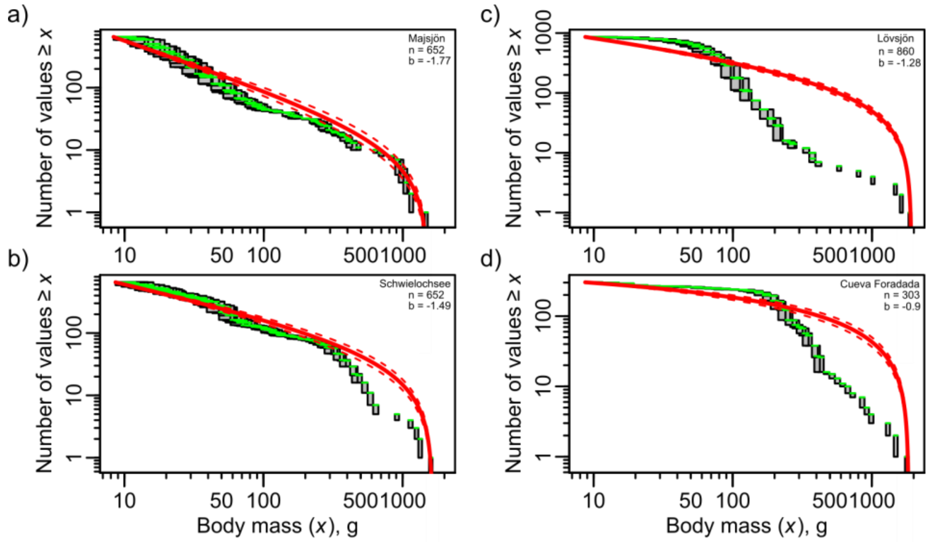

Figure S2 Here we show examples of two lakes with a relatively good MLE fit (a and b), and two lakes with a relatively ill MLE fit (c and d). In these plots we show the individual size distribution and MLE (bins) fit (red solid line), with 95% CI intervals (red dashed line). The horizontal green line shows the range of body sizes for each bin, with its value on the *y*-axis corresponding to the total number of individuals in bins whose minima are ≥ the bin's minimum. The vertical span of each grey bar shows the possible range of the number of individuals with body mass ≥ the body mass of individuals in that bin (its horizontal span is the same as for the green lines) (according to Edwards et al. (2020)). In the "ill-fitting" graphs one can see that there is an underestimation of individuals in the "small fish" (before the intersect of the red MLE curve with the data), and an overestimation of "large fish" (after the intersect of the red MLE curve with the data). Note that both y and x axes are displayed on a log scale, which is a similar depiction of how traditional SS slopes are displayed.

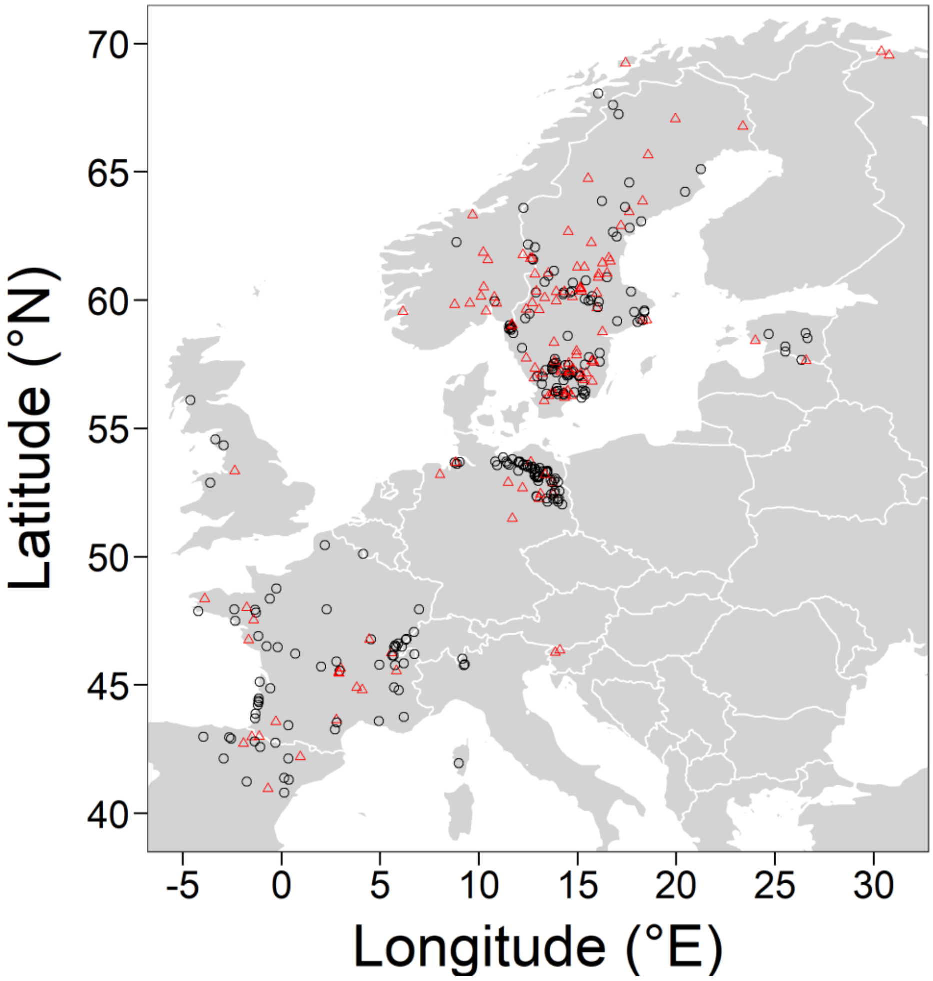

Figure S3 Map showing the distribution of lakes in our study, including lakes with a “good fit” (black circles) as well as lakes with a “bad fit” which were excluded from analyses (red triangles).

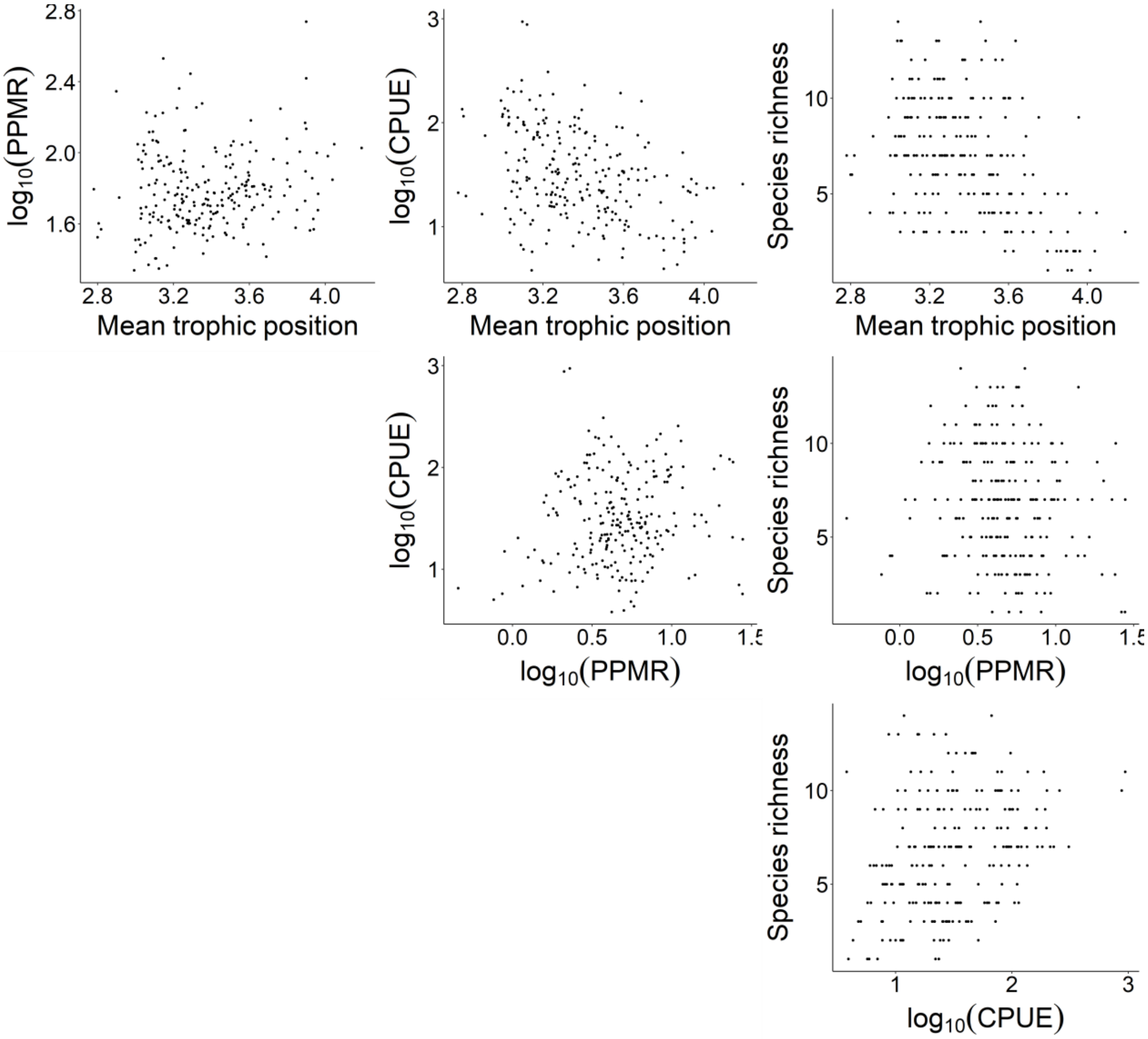

Figure S4 Correlation plots of the biotic variables (trophic position, $\log_{10}$(PPMR), $\log_{10}$(CPUE) and species richness). N=235 lakes.

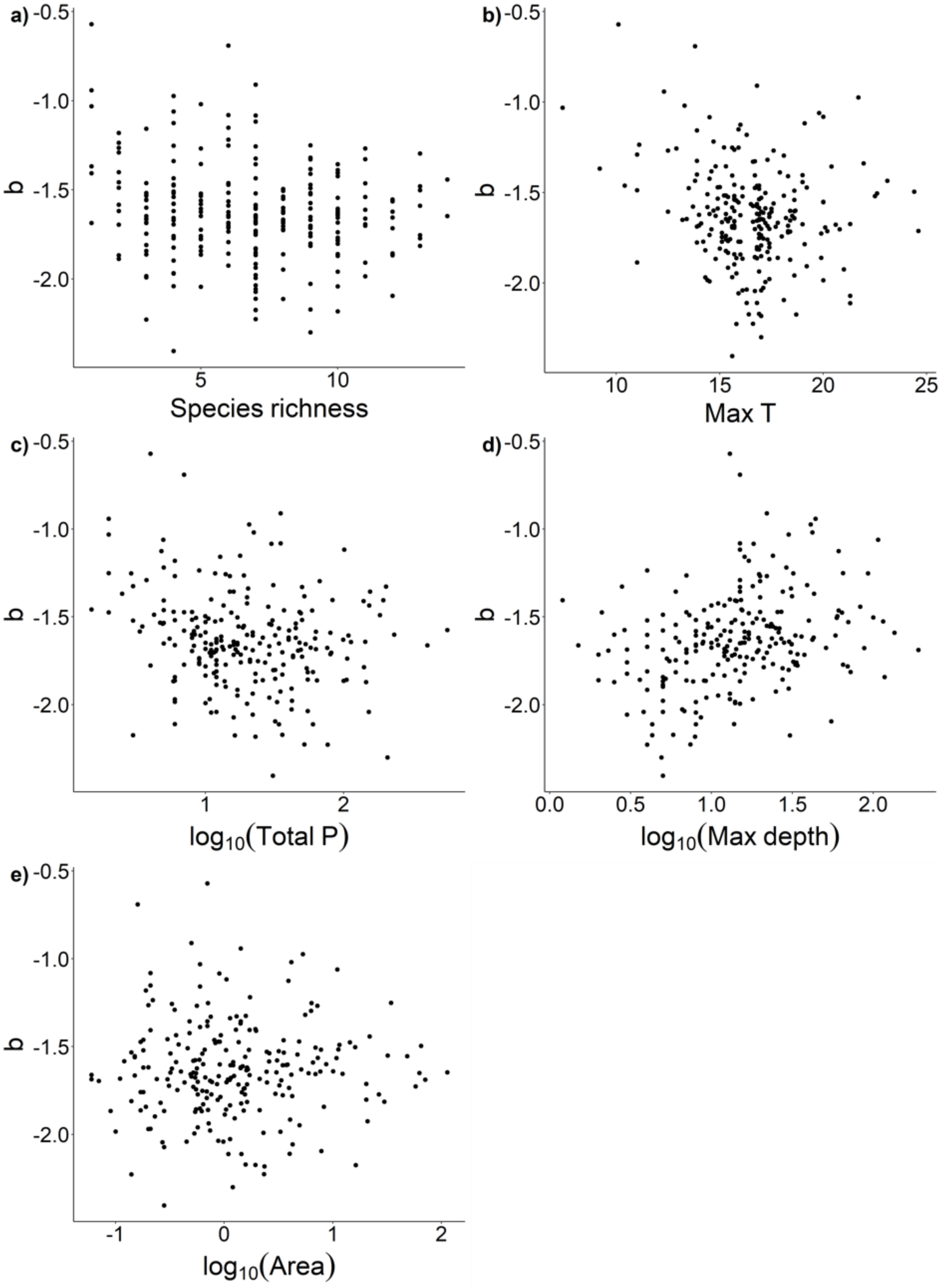

Figure S5 Marginal effect plots between the exponent b of the size spectrum and a) the species richness, b) the maximum temperature, c) total Phosphorus (log10), d) maximum depth (log10) and e) area (log10). The lines are significant regression lines with 95% CI intervals. Model outputs are shown in Table 3. N=235 lakes

Sensitivity analyses with fish mass 8-1000g (instead of 8-2000 g)

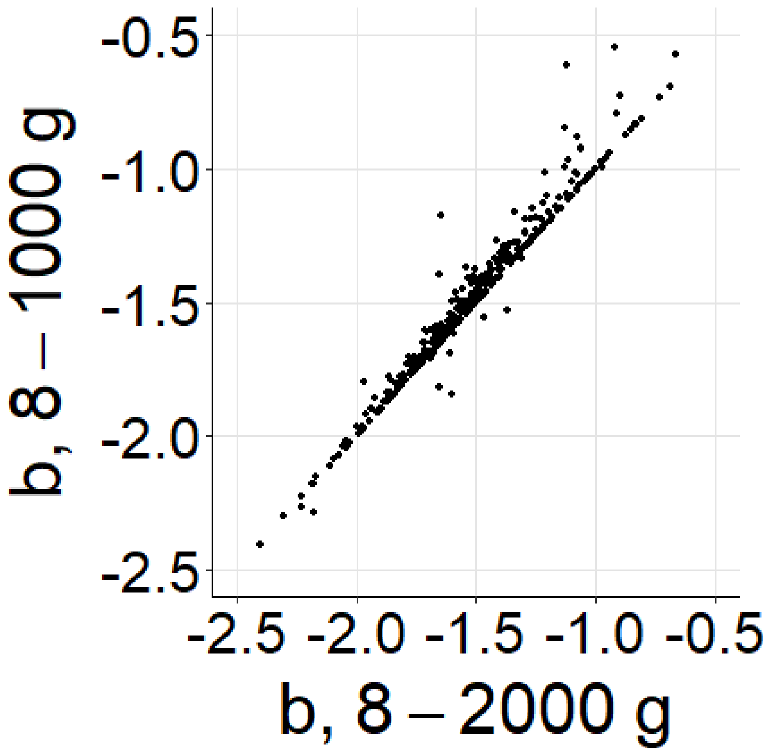

Figure S6 Comparison of exponent b calculated with fish with weight 8-2000 g (main analyses) and with weight 8-1000 g.

Table S3 Fish size 8-1000 g. Output of the model (linear mixed model with a structure to account for potential spatial autocorrelation) relating the exponent b of the size spectrum to the mean trophic position of the community, PPMR, CPUE, the species richness and four environmental covariates (maximum temperature, total phosphorus, maximum depth and lake area). In the last two columns standardized values and errors are noted. R^2 of the model is 0.40. N = 235 lakes. Significance codes: *** p < 0.001; ** p < 0.01; * p < 0.05; + p<0.1.

	**Value**	**Std. error**	**DF**	**t-value**	**p-value**	**Std. value**	**Std. error**
(Intercept)	-0.745	0.324	226	-2.300	0.022*	0.223	0.216
Trophic position	0.075	0.064	226	1.162	0.247	0.072	0.062
log_{10}(PPMR)	-0.429	0.051	226	-8.394	<0.001***	-0.404	0.048
log_{10}(CPUE)	-0.300	0.047	226	-6.437	<0.001***	-0.453	0.070
Species richness	-0.008	0.007	226	-1.176	0.241	-0.088	0.075
Max T	-0.012	0.010	226	-1.275	0.204	-0.103	0.081
log_{10}(Total P)	-0.007	0.048	226	-0.149	0.882	-0.012	0.077
log_{10}(Max depth)	-0.061	0.052	226	-1.177	0.241	-0.084	0.071
log_{10}(Area)	0.050	0.028	226	1.781	0.076+	0.110	0.062
